# Mapping of Nematode Resistance in Hexaploid Sweetpotato Using a Next-Generation Sequencing-Based Association Study

**DOI:** 10.3389/fpls.2022.858747

**Published:** 2022-03-18

**Authors:** Nozomi Obata, Hiroaki Tabuchi, Miyu Kurihara, Eiji Yamamoto, Kenta Shirasawa, Yuki Monden

**Affiliations:** ^1^Graduate School of Environmental and Life Science, Okayama University, Okayama, Japan; ^2^Kyusyu Okinawa Agricultural Research Center, National Agriculture and Food Research Organization, Miyakonojo, Japan; ^3^Faculty of Agriculture, Okayama University, Okayama, Japan; ^4^Graduate School of Agriculture, Meiji University, Kawasaki, Japan; ^5^Department of Frontier Research and Development, Kazusa DNA Research Institute, Kisarazu, Japan

**Keywords:** polyploidy, nematode, sweetpotato, resistant cultivar, breeding, association study

## Abstract

The southern root-knot nematode (SRKN; *Meloidogyne incognita*) is a typical parasitic nematode that affects sweetpotato [*Ipomoea batatas* (L.) Lam.], causing a significant decrease in crop yield and commercial value. In Japan, the SRKN is classified into 10 races: SP1–SP5, SP6-1, SP6-2, and SP7–SP9, with the dominant race differing according to the cultivation area. Soil insecticides have previously been used to reduce the soil density of SRKNs; however, this practice is both costly and labor intensive. Therefore, the development of SRKN-resistant sweetpotato lines and cultivars is necessary. However, due to the complexity of polyploid inheritance and the highly heterogeneous genomic composition of sweetpotato, genetic information and research for this species are significantly lacking compared to those for other major diploid crop species. In this study, we utilized the recently developed genome-wide association approach, which uses multiple-dose markers to assess autopolyploid species. We performed an association analysis to investigate resistance toward SRKN-SP2, which is the major race in areas with high sweetpotato production in Japan. The segregation ratio of resistant and susceptible lines in the F_1_ mapping population derived from the resistant “J-Red” and susceptible “Choshu” cultivars was fitted to 1: 3, suggesting that resistance to SP2 may be regulated by two loci present in the simplex. By aligning the double digest restriction-site associated DNA sequencing reads to the published *Ipomoea trifida* reference sequence, 46,982 single nucleotide polymorphisms (SNPs) were identified (sequencing depth > 200). The association study yielded its highest peak on chromosome 7 (Chr07) and second highest peak on chromosome 3 (Chr03), presenting as a single-dose in both loci. Selective DNA markers were developed to screen for resistant plants using the SNPs identified on Chr03 and Chr07. Our results showed that SRKN-SP2-resistant plants were selected with a probability of approximately 70% when combining the two selective DNA markers. This study serves as a model for the identification of genomic regions that control agricultural traits and the elucidation of their effects, and is expected to greatly advance marker-assisted breeding and association studies in polyploid crop species.

## Introduction

Sweetpotato [*Ipomoea batatas* (L.) Lam.], belonging to the Convolvulaceae family, is widely cultivated in both tropic and temperate zones. With an annual worldwide production of 91.8 million tons (FAOSTAT, 2019), it is regarded as the seventh most important crop species. Sweetpotato is rich in carbohydrates, vitamins (A, C, B1, B2, B3, B6, and E), biotin, dietary fiber, potassium, and other nutrients; thus, it plays an important role in food security, especially in developing countries. China is the world’s major producer of sweetpotato, followed by Sub-Saharan Africa. Approximately 52.0 million tons of sweetpotato are produced annually in China, accounting for approximately 56.6% of the total global production. Japan produces approximately 748,700 tons of sweetpotato annually and is the 17th largest producer in the world. In Japan, the Kagoshima and Miyazaki prefectures in the Kyushu region, and Ibaraki and Chiba prefectures in the Kanto region are the predominant production areas (Ministry of Agriculture, Forestry and Fisheries, 2020). In addition to being consumed as a raw food, sweetpotato is widely used in animal feed, processed foods, and the preparation of starch and alcohol. Thus, sweetpotato is a commercially important crop, and the development of cultivars with disease, pest, and nematode resistance is required to avoid commercial yield losses and expand the cultivation area.

The southern root-knot nematode (SRKN) (*Meloidogyne incognita*) is a typical nematode of sweetpotato, causing serious damage to the appearance, quality, and yield of the crop (Lawrence et al., 1986). Krusberg and Nielson (1958) reported that the yield of sweetpotato on farms with soil containing root-knot nematodes was 61% less than that on farms with pesticide-treated soil. These nematodes live in warm regions and have an optimum temperature of 25–30°C; furthermore, they have been detected in most areas where sweetpotato is cultivated (Yoshida, 1965; Iwahori et al., 2000; Iwahori and Sano, 2003). The host range of the SRKN is very wide, including thousands of agronomically important plants (Overstreet, 2009). When infested with the SRKN, host roots form humps (galls) with a diameter of 1–2 mm. This destroys and deforms the roots, inhibiting the absorption of nutrients and water, and leads to poor growth and death. In the roots of sweetpotato, dents occur when they are infested with SRKN. As the symptoms progress, constriction and dehiscence occur, resulting in a significant decrease in yield quality, and commercial value. The SRKN is classified into 10 races (SP1– SP5, SP6-1, SP6-2, SP7–SP9) in Japan (Sano and Iwahori, 2005; Tabuchi et al., 2017). Each race has a different geographical distribution. SP1 is mainly distributed in Saga, Kumamoto, and Nagasaki prefectures, and SP4 and SP6 races are predominantly distributed in Okinawa and also detected in Ibaraki and Chiba prefectures (Sano et al., 2002; Sano and Iwahori, 2005; Kuranouchi et al., 2018). SP2 is found mainly in the Kagoshima and Miyazaki prefectures, which have the highest levels of sweetpotato production in Japan. Treatment with insecticides such as D-D agents, although effective in controlling SRKN in the soil, is costly and labor-intensive (Krusberg and Nielson, 1958). Therefore, the development of sweetpotato cultivars with SRKN resistance is required.

Genetic studies in sweetpotato lag considerably behind those in other major diploid crop species owing to its complex mode of polyploid inheritance. Sweetpotato has a large genome (2.2–3 Gb) and is a hexaploid species with 90 chromosomes (2n = 6x = 90) (Isobe et al., 2017; Monden and Tahara, 2017). Although a few cultivars can reproduce among themselves, most show self-incompatibility or cross-incompatibility and are, therefore, genetically heterogeneous (Gurmu et al., 2013; Hirakawa et al., 2015). There is an ongoing debate as to whether sweetpotato is an autohexaploid or allohexaploid (Gao et al., 2020). Several early studies suggested that sweetpotato is allohexaploid (Ting and Kehr, 1953; Jones, 1967; Magoon et al., 1970; Sinha and Sharma, 1992). In contrast, more recent genetic analyses using molecular markers have suggested that it is autohexaploid, with some preferential pairing (Ukoskit and Thompson, 1997; Kriegner et al., 2003; Cervantes-Flores et al., 2008; Zhao et al., 2013; Monden et al., 2015). A recent study using ultra-dense multilocus genetic mapping also suggested that sweetpotato inheritance is highly autohexaploid-like, with random chromosome pairing enabling recombination between all homologous chromosomes during meiosis (Mollinari et al., 2020). Genetic studies have been successful in allopolyploid species due to their similarity to diploids in terms of segregation patterns and chromosomal pairing (Bourke et al., 2018; Mollinari and Garcia, 2019; da Silva Pereira et al., 2020; Yamamoto et al., 2020). However, these genetic approaches are unsuitable in autopolyploids, as they exhibit multiple heterozygous genotypes (Bourke et al., 2018; Yamamoto et al., 2020). Precise genetic mapping in autopolyploids is performed using multiple-dose markers wherein, the allele dosage for each marker needs to be determined (Mollinari et al., 2020; Yamamoto et al., 2020). However, the development of analytical tools for multiple-dose markers requires elaborate analytical algorithms, and this process has been challenging.

Genetic analyses using classical molecular markers such as random amplified polymorphic DNA (RAPD) and amplified fragment length polymorphism (AFLP) markers have identified DNA markers linked to SRKN resistance in sweetpotato (Ukoskit et al., 1997; Mcharo et al., 2005; Cervantes-Flores et al., 2008; Nakayama et al., 2012). Genetic analysis of SP1 and SP2 was performed using 92 lines from an F_1_ population derived from a cross between the resistant cultivar “Hi-Starch” and the susceptible cultivar “Koganesengan” (Nakayama et al., 2012). Bulked segregant analysis was performed to screen AFLP markers associated with SP1 and SP2 resistance, and interval mapping was conducted using the selected AFLP markers. As a result of interval mapping, a major quantitative trait locus (QTL) [here, named *qRmi (t)*] associated with resistance to SP1 and SP2 was detected. Furthermore, based on the AFLP markers in the QTL, a sequence characterized amplified region (SCAR) marker was developed to screen for resistant plants. Sasai et al. (2019) analyzed single nucleotide polymorphisms (SNPs) and retrotransposon insertion polymorphisms using next-generation sequencing (NGS) to construct a high-density genetic linkage map integrated with simple sequence repeat (SSR) markers. As a result of the QTL and genome-wide association study (GWAS) analyses, a major QTL common to SP1, SP4, and SP6-1 was identified, and a selective DNA marker that can easily and efficiently select resistant plants was developed. Additionally, Yamamoto et al. (2020) have developed a novel GWAS method for polyploid species that utilizes multiple-dose markers. With this method, the allele dosage of each marker can be determined using allele dosage probabilities calculated from the read counts of the NGS data. This technique has been shown as effective in the genetic analysis of autohexaploid sweetpotato.

In this study, we performed genetic mapping to identify the genomic regions controlling SRKN-SP2 resistance in sweetpotato. The aims of this study were as follows: (1) to identify a large number of genome-wide SNPs using double-digest restriction site-associated DNA sequencing (ddRAD-seq) analysis; (2) to conduct genetic mapping using the novel GWAS method and estimate the allele dosage probability for each SNP marker calculated on the basis of read depth information from the ddRAD-seq data; and (3) to develop highly selective DNA markers for SRKN resistance based on the DNA sequence of the identified genomic regions. This study presents an effective method to identify genomic regions that control agronomically important traits in sweetpotato and will help to guide future genetic mapping in autohexaploid crop species.

## Materials and Methods

### Plant Materials

The sweetpotato cultivars “J-Red” and “Choshu,” and an F_1_ mapping population consisting of 107 lines obtained by crossing these two cultivars were used in this study. “J-Red” was developed by crossing a high-starch, disease-resistant “Shiroyutaka” cultivar as the female parent, and a high carotene “86J-6” cultivar introduced from the United States, as the male parent. J-Red is resistant to all SRKN races except SP8 (Sano and Iwahori, 2005; Tabuchi et al., 2017). “Choshu” is a domestic cultivar that is susceptible to the SRKN races, SP1, SP2, SP3, SP4 SP6-1, and SP6-2 (Tabuchi et al., 2017). Genomic DNA was extracted from all plants using the DNeasy Plant Mini Kit according to the manufacturer’s instructions (QIAGEN, Hilden, Germany). The yield and quality of the extracted DNA was confirmed using a NanoDrop 2000 instrument (Thermo Fisher Scientific, Wilmington, DE, United States).

### Resistance Evaluation

Southern root-knot nematode resistance was evaluated at the National Agriculture and Food Research Organization, Kyusyu Okinawa Agricultural Research Center (Miyakonojo City, Miyazaki Prefecture). The resistance evaluation test was performed using second-stage juveniles (J2) of *M. incognita* according to the method developed by Tabuchi et al. (2017), which is originally described by Sano and Iwahori (2005). The juveniles of *M. incognita* were freshly prepared for each test as follows: A 19-day-old seedling of a susceptible tomato cultivar (Plitz) was inoculated in a 15 cm diameter pot containing approximately 600 g of a seeding culture soil (Kenbyo, Yaenogei, Japan) with approximately 6,000 J2 *M. incognita*, which remained from the former resistance test. Plitz plants were cultivated in a greenhouse at an average temperature of 25.5°C for 41–48 days. The egg masses, formed by the nematodes on the tomato root systems during cultivation, were picked up and placed on a cotton filter partially submerged in water in a beaker at 24°C. In this system, the J2 emerged from the egg masses, migrated through the filter, and accumulated at the bottom of the beaker. The cotton filter was transferred to a new beaker every 2–3 days, and the previous beaker was kept at 13°C, which is close to the developmental zero point for *M. incognita* (Gotoh et al., 1973). J2 individuals were collected from three to five beakers and used for a new resistance test.

Seedlings from each of the 107 lines of the F_1_ sweetpotato population and their parental cultivars were rooted in a greenhouse at an average temperature of 25.5^°^C, and after 7–10 days, a single node was collected by cutting approximately 1.5 cm of the vine. Two to six 9 cm pots containing approximately 200 g of mixed soil (Kenbyo:steam-sterilized andosol, 1:1) were prepared for each line, and a single node cutting was planted. After 3–4 days, 500 J2 (SP2) nematodes were inoculated into each pot. Each pot was covered with a newspaper for the first 3 days after inoculation. After 35 days, the roots were washed, egg masses were stained with a 0.02% erioglausin solution, and the number of egg masses were counted. As described previously (Sasai et al., 2019), we carried out resistance tests for at least four plans in two replications (two plants per replication). When the results of resistance or susceptibility were consistent among plants, the resistance test of the F_1_ line was considered complete. When the results were different, we continued resistance tests to obtain more accuracy. Finally, the resistance of F_1_ progeny was tested on 2–9 replications (4–18 plants for each line), and the calculated average number of egg masses was used to determine resistance. We used the number of egg masses per plant instead of eggs masses per gram of root for the following reasons: (1) In farmer’s fields, the number of egg masses per plant seems to be more important than egg masses per gram of root. (2) The number of egg masses per plant is more stable than that per gram of root because the coefficient of variation of the former is less than that of the latter in over 1,000 times resistance tests (unpublished data). (3) Both values show high correlation (>0.92 unpublished data). Following previous studies (Nakayama et al., 2012; Tabuchi et al., 2017; Sasai et al., 2019), we determined that plants with fewer than 10 egg masses per plant were resistant, and those with more than 10 egg masses were susceptible. The number of resistant and susceptible plants were analyzed, and a Chi-square goodness-of-fit test was performed with the calculated and expected segregation ratios using Microsoft excel.

### Genotyping With Double-Digest Restriction Site-Associated DNA Sequencing and Single Nucleotide Polymorphisms Calling

Genotyping was performed using ddRAD-seq according to previously described methods (Shirasawa et al., 2017; Sasai et al., 2019). Genomic DNA was digested with *Msp*I and *Pst*I restriction enzymes (FastDigest Enzymes, Thermo Fisher Scientific, Waltham, MA, United States), and adaptor ligation and purification were performed to prepare the ddRAD-seq library (Shirasawa et al., 2017). The ddRAD-seq library was sequenced using HiSeq4000 and NextSeq500 systems (Illumina, San Diego, CA, United States) and DNBSEQ-G400 (MGI, Shenzhen, China). The paired-end short reads with a read length of 150 bp were analyzed using the following procedure: first, they were trimmed based on the quality score (QV > 30) and the adapter sequence (5’-AGATCGGAAGAGC-3’) was removed using Cutadapt (version 2.8) (Martin, 2011). During this process, the minimum read length was set to 30 bases to obtain high-quality reads. Due to the absence of a pseudomolecule-level reference genome sequence for cultivated hexaploid sweetpotato (*I. batatas*), reads were then aligned to the whole genome sequence of *I. trifida* (Wu et al., 2018) using Bowtie2 (version 2.3.0) software (Langmead and Salzberg, 2012). The parameters were set to “–very-sensitive-local” mode for alignment. SNP calling was performed using the “mpileup” option in SAMtools v. 0.1.19 (Li et al., 2009) and the mpileup2snp option of VarScan 2 v.2.3 (Koboldt et al., 2012). The ddRAD-seq reads were under the accession number DDBJ: DRA013144.

### Allele Dosage Estimation and Association Analyses

The allele dosage estimation and association analyses were performed in accordance with an analytical method detailed in Yamamoto et al. (2020). Briefly, this method estimates allele dosage of polyploids by calculating the allele dosage probability using read count information in the NGS data. Here, allele dosage refers to the dosage of the reference genome-type allele for each SNP locus. For hexaploid species, the possible allele dosage states were 0/6 (aaaaaa), 1/6 (Aaaaaa), 2/6 (AAaaaa), 3/6 (AAAaaa), 4/6 (AAAAaa), 5/6 (AAAAAa), and 6/6 (AAAAAA). For the allele dosage estimation, the produced VCF file was loaded into the R platform *via* “read.vcfR” in vcfR (version 1.12.0) (Knaus and Grünwald, 2017). Information on the depths of the total (DP) and reference type (RD) reads were extracted from the VCF file using “extract.gt” in vcfR. High read depth is required for the accurate genotyping of polyploid species when compared with diploids. For example, a DP of 60–80 was recommended to distinguish between the genotypes for tetraploid species (AAAA, AAAa, AAaa, Aaaa, aaaa) (Uitdewilligen et al., 2013; Endelman et al., 2018). In this study, the minimum DP was set to 200 for accurate genotyping in the hexaploid sweetpotato; thus, individual genotypes with DP < 200 and DP > 1,000 were filtered out from further analyses. In addition, markers with missing values > 0.5 and major genotype frequency (MGF) > 0.95 were filtered out.

For the allele dosage estimation, we used the naïve method. The probability (Pr) of dosage for a given DP and RD was calculated using the binomial distribution function, as follows:


Pr⁡(Dosage=di)=CR⁢DD⁢P×riR⁢D×(1-ri)D⁢P-R⁢D

where *r*_*i*_ is the theoretical value of the allele dosage *d* (i.e., 0/6–6/6) in the individual *i*. The relative probability (RPr) of the reference type allele dosage for each SNP marker was calculated using the following formula:


$$\text{RPr}\left(\text{Dosage}=d_{i}\right)=\mathrm{Pr}\left(\text{Dosage}=d_{i}\right)/\sum_{i}\mathrm{Pr}\left(\text{Dosage}=d_{i}\right)$$

The calculation was performed using the R script “alleleDosageEstimation.R.” In the actual data, the theoretical probabilities *r* may deviate owing to errors caused in the experimental procedure. Therefore, the probability of allele dosage in the real data was calculated by including an unknown error probability of 0.001 using the option “read.err.prob” in “alleleDosageEstimation.R.” A matrix was obtained for each SNP marker, with individuals and the relative probabilities of the reference allele dosage calculated by the above equation as row and column elements, respectively.

The association study was performed in R (version 3.6.2) using the R script “alleleDosageGLM.R.” The number of egg masses was standardized by logarithmic transformation. After adding 1 to the average number of egg masses in each line, the resistance score was calculated by taking the common logarithm and used for GWAS. The four F_1_ lines JC5, JC22, JC57, and JC119 were excluded from further analyses because their resistance scores were not stable. In addition, JC2 was also excluded from further analyses because its genomic DNA was not available for genotyping with ddRAD-seq. Therefore, the F_1_ population used for the GWAS included 102 lines. The association between marker genotypes and phenotypes was investigated using a generalized linear model (GLM). GLM fitting was performed using “glm” in R (R Core Team, 2020). The augmented family functions “binomial” and “gaussian” were used for binary and continuous traits, respectively. The likelihood-ratio test for the SNP effect was performed using “pchisq” in R (R Core Team, 2020) with deviance and degrees of freedom from each GLM as arguments. The R package used in the analysis in this study is available at https://github.com/yame-repos/ngsAssocPoly. Statistical testing was performed using a Wilcoxon rank sum test. The “Wilcox.exact” function was used in the R package “exactRankTests.”

### Development of Selective Markers

Selective markers were developed to distinguish between J-Red and Choshu types based on the target SNPs identified by the GWAS. We obtained the chromosomal positions of target SNPs in the diploid reference genome of *I. trifida* (Wu et al., 2018). PCR primers were designed according to a previously described method (Hayashi et al., 2004). In this method, the target SNP was used as the base at the 3’ end of the primer, and an artificial mismatch was inserted three bases upstream. When designing the primers, the bam files obtained by aligning the short reads of the parents with the reference sequence of *I. trifida* were visualized using an Integrative Genomics Viewer (IGV) (Robinson et al., 2011), and the sequence information around the target SNP was extracted. If the sequence around the target SNP was not sufficiently covered by the short reads of the ddRAD-seq, the sequence around the SNP was determined by Sanger sequencing after TA cloning of the amplicon with the TOPO TA cloning kit according to the manufacturer’s instructions (Invitrogen, Carlsbad, CA, United States). Genotyping was performed using primers designed based on the target SNPs. The primers for the positive control were designed based on the *SSII* gene. The PCR solution contained 10 ng of DNA, 1.0 μL of 10 × PCR Buffer, 0.8 μL of dNTPs (2.5 mM each), 0.25 units of TAKARA Taq Hot Start Version, and 0.2 μL each of the forward and reverse primers (50 μM), adjusted to a total volume of 10 μL. The reaction conditions were as follows: initial denaturation at 94°C for 2 min, followed by 30 cycles of denaturation at 94°C for 10 s, annealing at 60°C for 10 s, and extension at 72°C for 30 s. Amplified products were visualized using electrophoresis on a 1.5% agarose gel (BioRAD, Hercules, CA, United States).

## Results

### Phenotyping for Nematode Resistance

**FIGURE 1:**
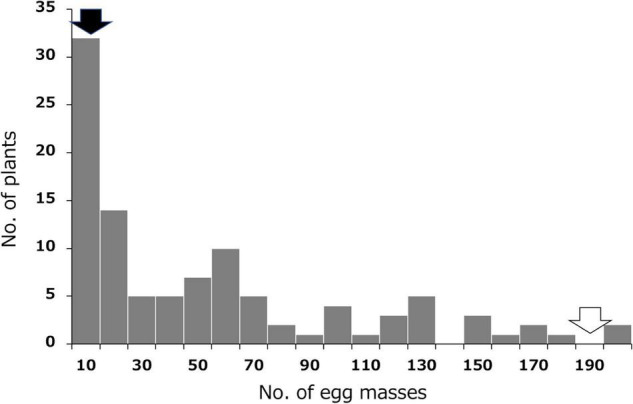
Frequency distribution for the mean number of SP2 *Meloidogyne incognita* egg masses in the F_1_ population derived from a cross between resistant “J-Red” and susceptible “Choshu” sweetpotato cultivars. Black and white arrows indicate the values of J-Red and Choshu, respectively.

Resistance to SRKN was evaluated by measuring the number of egg masses formed in the sweetpotato roots 35 days after inoculation with 500 nematodes. The results for the SRKN resistance tests in the F_1_ population and their parents are shown in Figure 1 and Supplementary Table 1. The average number of egg masses was 1.3 and 192.0 in the resistant parent “J-Red” and the susceptible parent “Choshu,” respectively, which indicated that there was a large difference in the resistance between the parental cultivars. The results of the frequency distribution of resistance in the F_1_ population showed a few lines with fewer egg masses than “J-Red” and a few lines with more egg masses than “Choshu,” and transgressive segregation was observed. The threshold for the number of egg masses that distinguishes resistance and susceptibility was set to 10 with previous studies (Nakayama et al., 2012; Tabuchi et al., 2017; Sasai et al., 2019), and the number of resistant and susceptible lines in the F_1_ population was determined to be 32 and 71, respectively. The ratio of the number of resistant and susceptible F_1_ lines corroborated with the expected 1:3 segregation ratio (χ^2^ = 2.023, *P* = 0.155). In hexaploid species, if one parent has a genotype with two dominant loci of the simplex allele (AaaaaaBbbbbb) and the other parent has a nullplex (aaaaaabbbbbb) genotype, “AaaaaaBbbbbb,” “Aaaaaabbbbbb/aaaaaaBbbbbb” and “aaaaaabbbbbb” genotypes were expected to segregate at a ratio of 1:2:1 in the F_1_ progeny. Therefore, the genotypes with both dominant loci (AaaaaaBbbbbb) and other genotypes (Aaaaaabbbbbb or aaaaaaBbbbbb or aaaaaabbbbbb) were expected to segregate at a ratio of 1:3 in the F_1_ progeny. Here, since the segregation ratios of resistant and susceptible F_1_ lines were fitted to 1:3, resistance to SP2 should be regulated by two loci present in a simplex allele.

### Association Analyses

**FIGURE 2:**
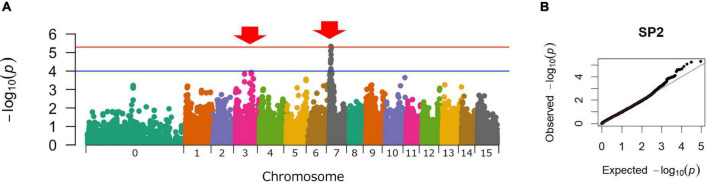
Manhattan and Q-Q plots for sweet potato resistance to SP2 *Meloidogyne incognita.* **(A)** Manhattan plot. *X*-axis: the chromosomal number; *Y*-Axis: -log_10_(P). The red line represents the significance threshold from the false Discovery rate (FDR) based on the Benjamini–Hochberg procedure (BH) (Benjamini and Hochber, 1995). The blue line represents the significance threshold −log_10_(1 × 10^−4^). **(B)** Q-Q plot.

Genome-wide SNPs were identified using the ddRAD-seq library, which generated a total of 1,887 million reads. After preprocessing, 1,771 million high-quality reads were obtained. The number of reads obtained from the parental cultivars and F_1_ lines is shown in Supplementary Table 2. The high-quality reads were aligned to the whole genome sequence of *I. trifida* using Bowtie 2 software and produced an average alignment rate of 94.6% for all plants. The alignment rates in the parental cultivars and F_1_ lines are shown in Supplementary Table 3. A total of 406,720 SNPs was detected using Varscan. The average read depth at this point was 53.9. In the present study, the minimum DP was set to 200 for the accurate genotyping of hexaploid sweetpotato. The allele dosage probability for each marker was calculated based on the read depth information from the ddRAD-seq genotyping data. After filtering the SNPs with the minimum DP set to 200, the average DP of the SNPs was 224.4. GWAS was performed using the remaining 46,788 SNPs. As a result, the highest peak was detected on chromosome 07 (Chr07), and the second highest peak was detected on chromosome 03 (Chr03) (Figure 2).

### Investigating the Effects of the Identified Loci

**FIGURE 3:**
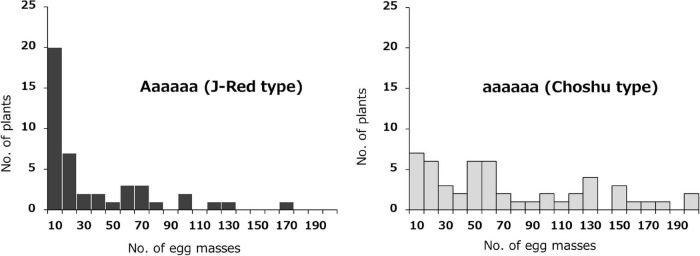
Frequency distribution of the mean number of egg masses on F_1_ lines grouped by genotype, based on the Chr07_1778460 SNP. Histograms with black and light gray indicate J-Red and Choshu genotype plants, respectively.

**FIGURE 4:**
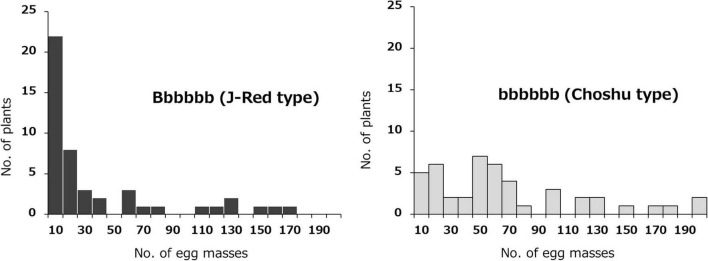
Frequency distribution of the mean number of egg masses on F_1_ lines grouped by genotype, based on the Chr03_18847788 SNP. Histograms with black and light gray indicate J-Red and Choshu genotype plants, respectively.

The effects of the two identified loci (on Chr03 and Chr07) on SRKN resistance were examined. The SNP marker for Chr07_1778460 was most associated with resistance on chromosome 7 (*P* = 4.671E-06). In the Chr07_1778460 marker, the resistant parent “J-Red” and the susceptible parent “‘Choshu” showed the genotypes of Aaaaaa (simplex) and aaaaaa (nullplex), respectively (Figure 3). Here, “A” represents a nucleotide that is identical with that of the reference sequence, while “a” represents a nucleotide that is different from that of the reference sequence. The total number of F_1_ lines used to investigate the effects of the identified loci was 102 since five lines were not available (see section “Materials and Methods”). Among the F_1_ population, the genotypes of eight lines with DP values < 200 were not determined. As a result, the F_1_ mapping population was classified into 43 and 51 lines for the J-Red (Aaaaaa) and Choshu (aaaaaa) types, respectively (Supplementary Table 4). In the F_1_ mapping population, the lines with the J-Red genotypes were distributed on the resistance side (Figure 3), and 20 of these 43 lines (46.5%) showed resistance (<10 egg masses in the resistance test). The average number of egg masses was 30.8 and 69.3 in the J-Red and Choshu types, respectively, showing a significant difference at the 0.1% level between the two genotypes (*P* = 3.763E-05, Wilcoxon rank sum test; Supplementary Figure 1). Subsequently, the SNP marker for Chr03_18847788 was strongly associated with resistance on chromosome 3 (*P* = 1.209E-04). In the Chr03_18847788 marker, J-Red and Choshu showed the genotypes of Bbbbbb (simplex) and bbbbbb (nullplex), respectively. Here, “B” represents a nucleotide that is identical with that of the reference sequence, while “b” represents a nucleotide that is different from that of the reference sequence. In the F_1_ population, the genotypes of nine lines with DP values < 200 were not determined. As a result, the F_1_ mapping population was classified into 46 and 45 lines for the J-Red (Bbbbbb) and Choshu (bbbbbb) types, respectively (Supplementary Table 5). Only two lines (JC159 and JC132) showed genotypes that were unlikely to emerge from their parents. Similar to the marker on Chr07_1778460, the lines with the J-Red genotypes (Bbbbbb) were unevenly distributed on the resistance side (Figure 4). Among the 46 lines with the J-Red genotype, 22 lines (47.8%) showed resistance. The average number of egg masses was 33.5 and 64.3 for the J-Red and Choshu genotypes, respectively, showing a significant difference at the 0.1% level between the two genotypes (*P* = 1.530E-04, Wilcoxon rank sum test; Supplementary Figure 1).

**FIGURE 5:**
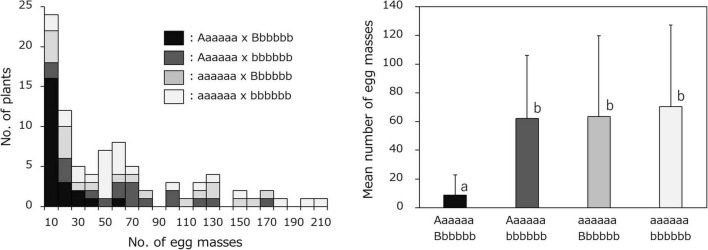
Frequency distribution of the mean number of egg masses on F_1_ lines grouped by genotype, based on the Chr07_1778460 (Aaaaaa or aaaaaa) and Chr03_18847788 (Bbbbbb or bbbbbb) SNPs. The F1 mapping population was divided into four groups: Aaaaaa × Bbbbbb, Aaaaaa × bbbbbb, aaaaaa × Bbbbbb, and aaaaaa × bbbbbb.

The combined effects of the two loci, Chr07_1778460 (Aaaaaa) and Chr03_18847788 (Bbbbbb) on resistance were then investigated. The F_1_ mapping population was divided into four groups: 23 lines with the genotype combination of Aaaaaa (J-Red type) and Bbbbbb (J-Red type), 18 lines with the genotype combination of Aaaaaa (J-Red type) and bbbbbb (Choshu type), 20 lines with the genotype combination of aaaaaa (Choshu type) and Bbbbbb (J-Red type), and 25 lines with the genotype combination of aaaaaa (Choshu type) and bbbbbb (Choshu type) (Figure 5 and Supplementary Table 6). The average number of egg masses for the lines in each genotype combination was 8.7, 62.1, 63.6, and 70.4, respectively (Figure 5). The number of egg masses on lines showing the J-Red type with both SNP markers was significantly lower than that of lines showing other genotype combinations (significant difference at the 0.1% level on Bonferroni-corrected Wilcoxon rank sum test; Figure 5). In addition, the average number of egg masses for Aaaaaa (J-Red type) and Bbbbbb (J-Red type) was 8.7, which was below the resistance threshold of 10. Among the 23 lines with the Aaaaaa (J-Red type) and Bbbbbb (J-Red type) genotype, 16 (69.6%) showed resistance. The rate of resistance was 46.5 and 47.8% for the J-Red type of Chr07_1778460 and Chr03_18847788 SNP markers, respectively. These results suggest that the lines with resistance against SP2 could be selected with a higher probability by combining two loci than by using a single locus.

### Development of Selective Markers

**FIGURE 6:**
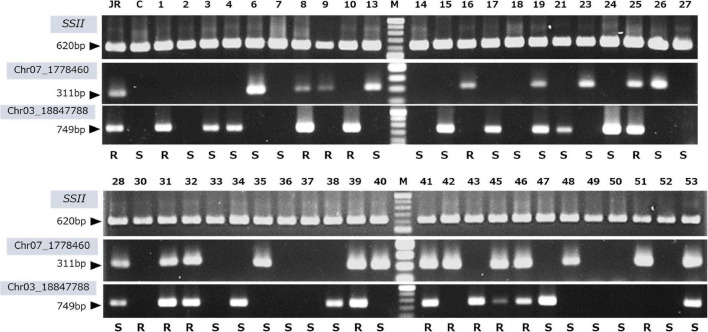
PCR genotyping using the developed DNA markers derived from the Chr07_1778460 and Chr03_18847788 SNPs. *SSII*, positive control; JR, J-Red; C, Choshu, 1-53, F_1_ lines; and M, 100 bp DNA ladder. The evaluation of the resistance to the SP2 race is shown on bottom of each lane. R and S indicates resistance and susceptibility, respectively.

J-Red derived (Chr07_1778460 and Chr03_18847788) SNPs were used to design genotyping PCR primers to assess SRKN-SP2 resistance. For designing PCR primers, we extracted the sequence information around the target SNPs based on the ddRAD-seq reads aligned to the *I. trifida* reference sequence. Since the sequence around the Chr07_1778460 SNP was not sufficiently covered by the short reads of the ddRAD-seq, the sequence around the SNP was determined by Sanger sequencing. The primer sequences are shown in Supplementary Table 7. A clear single band was observed in J-Red, but not in Choshu, for both Chr03 and Chr07 SNPs (Figure 6). Consequently, we used these primers to genotype the parental cultivars and 103 F_1_ lines (Figure 6 and Supplementary Figure 2). The total number of F_1_ lines used for genotyping with SNP-derived PCR primers was 103 because JC2 was available for this experiment. Using a marker designed to target the Chr03-derived SNP, the PCR product obtained was 749 bp. The presence or absence of the PCR band was 100% consistent with the genotype information. Of the 55 lines identified as J-Red type on the Chr03 SNP, 25 exhibited resistance (45.5%). Using a marker designed to target the Chr07-derived SNP, the PCR product obtained was 311 bp. As for Chr03, the presence or absence of the PCR band was 100% consistent with the genotype information. Of the 48 cultivars identified as J-Red type on the Chr07 SNP, 23 exhibited resistance (47.9%). Of the 27 cultivars that showed the J-red type at both Chr03 and Chr07 loci, 19 showed resistance (70.4%). Thus, it was shown that resistant plants could be selected with a high probability using two selective markers.

## Discussion

In this study, we assessed SRKN-SP2 resistance in sweetpotato using a novel GWAS method for polyploids based on allele dosage probability. Because the SRKN causes serious damage to the appearance, quality, and yield of sweetpotato, the development of SRKN-resistant cultivars/lines has been required. However, owing to the complexity of polyploid inheritance and the highly heterogeneous genomic composition of sweetpotato, genetic studies of this crop lag far behind those of major diploid crop species. The segregation ratio of resistant and susceptible lines in the F_1_ population suggested that resistance to SP2 is regulated by two loci in a single dose. GWAS analysis identified the two loci on Chr07 and Chr03, and selective DNA markers were developed based on the identified SNPs. Our results demonstrated that when two selective SNP-derived markers were used, resistant plants could be selected with a high probability (∼70%).

Since J-red shows resistance to the SRKN races SP1–9 (except SP8), it can be used as a resistant parental cultivar to identify the genetic regions associated with resistance for most SRKN races. In our previous study, we performed genetic analysis on SP1, SP4, and SP6-1 to identify the genetic regions controlling resistance and develop selective DNA markers (Sasai et al., 2019). For these three races, resistant and susceptible lines in the F_1_ population segregated in a 1:1 ratio. Therefore, it was suggested that one major QTL in simplex determined the resistance. As expected, one major QTL common to the three races present in the simplex was detected (Sasai et al., 2019). In contrast, for the SP2 race targeted in this study, the segregation ratio of resistance and susceptible lines in the F_1_ population was 1:3. This suggests that resistance to this race is regulated by two loci present in simplex. However, because the segregation ratio is close to 1:4, there is a possibility of regulation by one locus present in a duplex manner. Since the population size used in this study is relatively small for hexaploids (∼ 100 lines), it was difficult to clearly predict whether resistance to SP2 is controlled by two loci present in simplex or one locus present in duplex based on the segregation ratio. The association analysis showed that resistance to the SP2 race was controlled by two loci in simplex. For further confirmation, GWAS was performed using only simplex SNPs showing a simplex segregation ratio according to a previous method (Sasai et al., 2019). The segregation ratio of the SNPs in the F_1_ population was calculated, and SNPs fitted to the expected segregation ratio of the single dose were extracted based on a Chi-square goodness-of-fit test (*P* ≥ 0.01). Simplex markers that are present in one parent and are absent in the other parent segregate at a 1:1 ratio, and double simplex markers that are present in both parents segregate at a 1:3 ratio (Supplementary Table 8). GWAS was performed using the selected simplex SNPs with Tassel version 5.0 (Bradbury et al., 2007). The total number of SNPs selected to be present in simplex for both parents (double simplex) or simplex for one parent was 33,886 (Supplementary Table 8). GWAS using these SNPs generated peaks in Chr07 and Chr03 (Supplementary Figure 3). Because GWAS peaks were detected at the same loci using two completely independent analytical methods, the results of this study were considered reliable.

Several studies have presented genetic analyses on nematode resistance in sweetpotato. Nakayama et al. (2012) conducted genetic analysis of SP2 race using AFLP markers and developed SCAR markers associated with resistance. A major QTL [*qRmi(t)*] associated with resistance to SP1 and SP2 was detected. At that time, the reference genome for *I. trifida* had not been deciphered, and the chromosomal position of this QTL was unknown. However, we have determined that it is unlikely to be one of the QTLs identified in our study. The reasons for this are as follows: First, in the 2012 study, resistant and susceptible lines segregated in a 1:1 ratio in the F_1_ population of “Hi-starch” and “Koganesengan,” and one locus was identified. In contrast, in the present study, resistant and susceptible lines segregated in a 1:3 ratio, and two loci were identified. Second, according to Nakayama et al. (2012), the resistance to SP1 and SP2 in F_1_ was highly correlated (*r* = 0.8199), and a QTL common to SP1 and SP2 was detected, as expected. In contrast, the F_1_ population used in our study had a low correlation of resistance to SP1 and SP2 (*r* = 0.5681), and a single QTL was detected for SP1, whereas two QTLs were detected for SP2. When the primer sequences designed based on the QTL identified by Nakayama et al. (2012) were BLASTed to the reference sequence (*I. trifida*) used in our study, most of them showed no hits or hits on chromosome 12 (*E*-value < 1e-2). Hence, the genomic regions controlling resistance to SP2 race in the “Hi-Starch” and “J-Red” varieties are probably different.

The analytical method used in our study has the advantage of being relatively easy to use (Yamamoto et al., 2020). Currently, various analytical tools such as MAPpoly and QTLpoly have been developed for polyploid species, which use Mendelian inheritance simplex markers as well as multiplex markers (Mollinari and Garcia, 2019; da Silva Pereira et al., 2020; Mollinari et al., 2020). Recently, Oloka et al. (2021) performed QTL mapping for nematode resistance by constructing a high-density linkage map with single- and multiple-dose SNPs and indels. The authors utilized new bioinformatic tools developed for sweetpotato improvement, such as GBSpoly, a genotyping platform that modifies GBS to polyploid species; MAPpoly, an R package for building linkage maps; and QTLpoly, an R package for QTL mapping. A high-density integrated linkage map was developed using a mapping population derived from a cross between “Tanzania,” an African landrace cultivar, and “Beauregard,” a major United States cultivar. A major QTL for SRKN race 3 resistance was identified on linkage group 7 (LG7), and the genotypes of the most significant markers associated with resistance were duplex (AAaaaa) for the resistant “Tanzania” cultivar, and nulliplex (aaaaaa) for “Beauregard.” Oloka et al. (2021) used the same reference sequence of *I. trifida* (Wu et al., 2018) used in our study, and interestingly, one major peak was commonly detected on chromosome 7 (LG7) in both studies. However, it is unclear whether SRKN race 3 from Oloka et al. (2021) and SP3 used in this study are the same. In addition, the genetic distance between resistance cultivars “Tanzania” and “J-Red” is unknown. From these reasons, it cannot be determined whether the QTLs identified in these studies are consistent and/or derived from the same resistance gene. On the other hand, the analytical methods used in their study were useful for the identification of QTLs and investigation of their effects in hexaploid sweetpotato. However, the analytical tool used in our study is easy to use and can detect target regions without constructing linkage maps, and the obtained results are highly reliable. In general, linkage map construction for polyploid species requires considerable time and computational resources. In addition, when the size of the mapping population is relatively small, it is extremely difficult to accurately calculate the recombination values and marker distances. In contrast, the GWAS method used in this study can identify the genomic regions controlling agronomical traits in a short time (several minutes), even with limited computer resources. Although the loci identified in this study were in a single dose, this technique can also be used to identify loci in multiplexes. Therefore, it was determined that the method used in our analysis can effectively detect QTLs and be used to develop DNA markers in polyploid crop species.

The purpose of plant breeding is to produce excellent cultivars, which requires continuous discovery of desirable genes and pyramiding into breeding lines. Molecular markers linked to a gene or loci of target traits have been effectively utilized in breeding programs for cultivar development. In particular, the importance of molecular markers associated with pest and disease resistance is widely recognized, and such markers facilitate the development of resistant cultivars/lines by marker-assisted selection, which contributes to expanded cultivation areas and increased yields. In this study, the selective DNA markers were developed using the SNPs derived from J-Red resistant cultivar. Using the two selective markers, we could screen resistant lines to SRKN SP2 race with high efficiency (∼70%). However, the utility and effectiveness of these selective markers in other mapping populations and cross combinations have not been validated. In the near future, we aim to perform marker genotyping and resistance evaluation using other mapping populations and various sweetpotato cultivars/lines to validate the effectiveness and utility of these selective markers. In addition, we plan to perform comparative analysis with other tools, such as MAPpoly and QTLpoly, and identify candidate genes using RNA-sequencing analysis and transgenic experiments. Overall, this study provides a model for identifying genomic regions controlling agronomical traits and their effects in polyploid crop species. Our study also serves as a resource describing useful molecular tools for marker-assisted selection in sweetpotato breeding programs.

## Data Availability Statement

The datasets presented in this study can be found in online repositories. The names of the repository/repositories and accession number(s) can be found in the article/Supplementary Material.

## Author Contributions

YM and HT conceived and designed the experiments. NO, MK, HT, EY, KS, and YM contributed to the experiments and data analyses. NO, HT, and YM drafted the manuscript. All authors read and approved the final manuscript.

## Conflict of Interest

The authors declare that the research was conducted in the absence of any commercial or financial relationships that could be construed as a potential conflict of interest.

## Publisher’s Note

All claims expressed in this article are solely those of the authors and do not necessarily represent those of their affiliated organizations, or those of the publisher, the editors and the reviewers. Any product that may be evaluated in this article, or claim that may be made by its manufacturer, is not guaranteed or endorsed by the publisher.

## References

[B1] Benjamini Y. Hochberg Y. (1995). Controlling the false discovery rate: a practical and powerful approach to multiple testing. *J. R. Stat. Soc. Ser. B. Methodol.* 57, 289–300. 10.1111/j.2517-6161.1995.tb02031.x

[B2] Bourke P. M. Voorrips R. E. Visser R. G. F. Maliepaard C. (2018). Tools for Genetic Studies in Experimental Populations of Polyploids. *Front. Plant Sci.* 9:513. 10.3389/fpls.2018.00513 29720992 PMC5915555

[B3] Bradbury P. J. Zhang Z. Kroon D. E. Casstevens T. M. Ramdoss Y. Buckler E. S. (2007). TASSEL: software for association mapping of complex traits in diverse samples. *Bioinformatics* 23 2633–2635. 10.1093/bioinformatics/btm308 17586829

[B4] Cervantes-Flores J. C. Yencho G. C. Pecota K. V. Sosinski B. Mwanga R. O. M. (2008). Detection of quantitative trait loci and inheritance of root-knot nematode resistance in sweetpotato. *J. Am. Soc. Hortic. Sci.* 133 844–851. 10.21273/JASHS.133.6.844

[B5] da Silva Pereira G. Gemenet D. C. Mollinari M. Olukolu B. A. Wood J. C. Diaz F. (2020). Multiple QTL mapping in autopolyploids: a random-effect model approach with application in a hexaploid sweetpotato full-sib population. *Genetics* 215 579–595. 10.1534/genetics.120.303080 32371382 PMC7337090

[B6] Endelman J. B. Carley C. A. S. Bethke P. C. Coombs J. J. Clough M. E. da Silva W. L. (2018). Genetic variance partitioning and genome-wide prediction with allele dosage information in autotetraploid potato. *Genetics* 209 77–87. 10.1534/genetics.118.300685 29514860 PMC5937173

[B7] FAOSTAT (2019). *Food and Agriculture Organization of the United Nations (FAO).* Available online at: https://www.fao.org/faostat/en/#home (accessed August 4, 2021).

[B8] Gao M. Soriano S. F. Cao Q. Yang X. Lu G. (2020). Hexaploid sweetpotato (Ipomoea batatas (L.) Lam.) may not be a true type to either auto- or allopolyploid. *PLoS One* 15:e0229624. 10.1371/journal.pone.0229624 32126067 PMC7053752

[B9] Gotoh A. Sano Z. Minagawa N. (1973). Prediction of the time of emergence of the next generation of the southern root-knot nematode, Meloidogyne incognita, with thermal contents for development from infection populations. *Kyushu Plant Prot. Res.* 19 124–127.

[B10] Gurmu F. Hussein S. Laing M. (2013). Self-and cross-incompatibilities in sweetpotato and their implications on breeding. *Aust. J. Crop Sci.* 7:2074.

[B11] Hayashi K. Hashimoto N. Daigen M. Ashikawa I. (2004). Development of PCR-based SNP markers for rice blast resistance genes at the Piz locus. *Theor. Appl. Genet.* 108 1212–1220. 10.1007/s00122-003-1553-0 14740086

[B12] Hirakawa H. Okada Y. Tabuchi H. Shirasawa K. Watanabe A. Tsuruoka H. (2015). Survey ofgenome sequences in a wild sweet potato, Ipomoea trifida (H. B. K.) G. Don. *DNA Res.* 22 171–179. 10.1093/dnares/dsv002 25805887 PMC4401327

[B13] Isobe S. Shirasawa K. Hirakawa H. (2017). Challenges to genome sequence dissection in sweetpotato. *Breed. Sci.* 67 35–40. 10.1270/jsbbs.16186 28465666 PMC5407923

[B14] Iwahori H. Sano Z. (2003). Distribution of main plant-parasitic nematodes in sweet potato and taro fields in Kyushu and Okinawa, Japan. 3. Survey in the northern part in Kyushu Island (Fukuoka, Saga, Nagasaki and Ohita Prefs.). *Kyushu Plant Prot. Res.* 49 83–87. 10.4241/kyubyochu.49.83

[B15] Iwahori H. Sano Z. Ogawa T. (2000). Distribution of main plant-parasitic nematodes in sweet potato and taro fields in Kyushu and Okinawa, Japan. 1. Survey in the central and southern parts in Kyushu Island (Kumamoto, Miyazaki and Kagoshima Prefs.) and development of an effective DNA analysis method for species identification. *Kyushu Plant Prot. Res.* 46 112–117. 10.4241/kyubyochu.46.112

[B16] Jones A. (1967). *Theoretical Segregation Ratios of Qualitatively Inherited Characters for Hexaploid Sweet Potato (Ipomoea batatas L.). Technical Bulletin No. 1368.* Washington: US Department of Agriculture, Economic Research Service, 1–6.

[B17] Knaus B. J. Grünwald N. J. (2017). VCFR: a package to manipulate and visualize variant call format data in R. *Mol. Ecol. Resour.* 17 44–53. 10.1111/1755-0998.12549 27401132

[B18] Koboldt D. C. Zhang Q. Larson D. E. Shen D. McLellan M. D. Lin L. (2012). VarScan 2: somatic mutation and copy number alteration discovery in cancer by exome sequencing. *Genom. Res.* 22 568–576. 10.1101/gr.129684.111 22300766 PMC3290792

[B19] Kriegner A. Cervantes J. C. Burg K. Mwanga R. O. M. Zhang D. (2003). A genetic linkage map of sweet potato [Ipomoea batatas (L.) Lam.] based on AFLP markers. *Mol. Breed.* 11 169–185. 10.1023/A:1022870917230

[B20] Krusberg L. R. Nielson L. W. (1958). Pathogenesis of root-knot nematodes to the Port Rico variety of sweetpotsto. *Phytopathology* 48 30–39.

[B21] Kuranouchi T. Momota Y. Takada A. Katayama K. (2018). The composition of southern root-knot nematode (Meloidogyne incognita) race in the resistance test field used for sweetpotato breeding. *Nematol. Res.* 48 31–34. 10.3725/jjn.48.31

[B22] Langmead B. Salzberg S. L. (2012). Fast gapped-read alignment with Bowtie 2. *Nat. Meth.* 9 357–359. 10.1038/nmeth.1923 22388286 PMC3322381

[B23] Lawrence G. W. Clark C. A. Wright V. L. (1986). Influence of Meloidogyne incognita on resistant and susceptible sweet potato cultivars. *J. Nematol.* 18 59–65. 19294141 PMC2618494

[B24] Li H. Handsaker B. Wysoker A. Fennell T. Ruan J. Homer N. (2009). The sequence alignment/map format and SAMtools. *Bioinformatics* 25 2078–2079. 10.1093/bioinformatics/btp352 19505943 PMC2723002

[B25] Magoon M. L. Krishnan R. Vijaya Bai K. (1970). Cytological evidence on the origin of sweet potato. *Theor. Appl. Genet.* 40 360–366. 10.1007/BF00285415 24435948

[B26] Martin M. (2011). Cutadapt removes adapter sequences from high-throughput sequencing reads. *EMBnet J.* 17:10. 10.14806/ej.17.1.200

[B27] Mcharo M. LaBonte D. R. Clark C. Hoy M. Oard J. H. (2005). Molecular marker variability for southern root-knot nematode resistance in sweetpotato. *Euphytica* 144 125–132. 10.1007/s10681-005-5271-3

[B28] Ministry of Agriculture, Forestry and Fisheries (2020). *The 94th Statistical Yearbook of Ministry of Agriculture, Forestry and Fisheries.* Available online at: https://www.maff.go.jp/e/data/stat/94th/ (accessed February 5, 2022).

[B29] Mollinari M. Garcia A. A. F. (2019). Linkage analysis and haplotype phasing in experimental autopolyploid populations with high ploidy level using hidden markov models. *G3* 9 3297–3314. 10.1534/g3.119.400378 31405891 PMC6778803

[B30] Mollinari M. Olukolu B. A. Pereira G. D. S. Khan A. Gemenet D. Yencho G. C. (2020). Unraveling the hexaploid sweet-potato inheritance using ultra-dense multilocus mapping. *G3* 10 281–292. 10.1534/g3.119.400620 31732504 PMC6945028

[B31] Monden Y. Hara T. Okada Y. Jahana O. Kobayashi A. Tabuchi H. (2015). Construction of a linkage map based on retrotransposon insertion polymorphisms in sweetpotato via high-throughput sequencing. *Breed. Sci.* 65 145–153. 10.1270/jsbbs.65.145 26069444 PMC4430505

[B32] Monden Y. Tahara M. (2017). Genetic linkage analysis using DNA markers in sweetpotato. *Breed. Sci.* 67 41–51. 10.1270/jsbbs.16142 28465667 PMC5407921

[B33] Nakayama H. Tanaka M. Takahata Y. Matsui K. Iwahori H. Sano Z. (2012). Development of AFLP-derived SCAR markers associated with resistance to two races of southern root-knot nematode in sweetpotato. *Euphytica* 188 175–185. 10.1007/s10681-012-0678-0

[B34] Oloka B. M. da Silva Pereira G. Amankwaah V. A. Mollinari M. Pecota K. V. Yada B. (2021). Discovery of a major QTL for root-knot nematode (Meloidogyne incognita) resistance in cultivated sweetpotato (Ipomoea batatas). *Theor. Appl. Genet.* 134 1945–1955. 10.1007/s00122-021-03797-z 33813604 PMC8263542

[B35] Overstreet C. (2009). “Chapter 9, Nematoses,” in *The Sweetpotato*, eds Loebenstein G. Thottappilly G. (Berlin: Springer).

[B36] R Core Team (2020). *R: A Language and Environment for Statistical Computing.* Vienna: R Foundation for Statistical Computing.

[B37] Robinson J. T. Thorvaldsdóttir H. Winckler W. Guttman M. Lander E. S. Getz G. (2011). Integrative genomics viewer. *Nat. Biotechnol.* 29 24–26. 10.1038/nbt.1754 21221095 PMC3346182

[B38] Sano Z. Iwahori H. (2005). Regional variation in pathogenicity of Meloidogyne incognita populations on sweet potato in Kyushu Okinawa, Japan. *JPN. J. Nematol.* 35 1–12. 10.3725/jjn1993.35.1_1

[B39] Sano Z. Iwahori H. Tateishi Y. Kai Y. (2002). Differences in the resistance of sweet potato cultivars and lines to Melodogyne incognita populations. *JPN. J. Nematol.* 32 77–86. 10.3725/jjn1993.32.2_77

[B40] Sasai R. Tabuchi H. Shirasawa K. Kishimoto K. Sato S. Okada Y. (2019). Development of molecular markers associated with resistance to Meloidogyne incognita by performing quantitative trait locus analysis and genome-wide association study in sweetpotato. *DNA Res.* 26 399–409. 10.1093/dnares/dsz018 31377774 PMC6796513

[B41] Shirasawa K. Tanaka M. Takahata Y. Ma D. Cao Q. Liu Q. (2017). A high-density SNP genetic map consisting of a complete set of homologous groups in autohexaploid sweetpotato (Ipomoea batatas). *Sci. Rep.* 7:44207. 10.1038/srep44207 28281636 PMC5345071

[B42] Sinha S. Sharma S. N. (1992). Taxonomic significance of karyo- morphology in Ipomoea spp. *Cytologia* 57 289–293. 10.1508/cytologia.57.289

[B43] Tabuchi H. Kuranouchi T. Kobayashi A. Monden Y. Kishimoto K. Tahara M. (2017). Southern root-knot nematode race SP6 is divided into two races. *Nematol. Res.* 47 29–33. 10.3725/jjn.47.29

[B44] Ting Y. C. Kehr A. E. (1953). Meiotic studies in the sweet potato (Ipomoea batatas Lam.). *J. Hered.* 44 207–211. 10.1093/oxfordjournals.jhered.a106395

[B46] Uitdewilligen J. G. A. M. L. Wolters A. M. D’Hoop B. B. Borm T. J. Visser R. G. van Eck H. J. (2013). A next-generation sequencing method for genotyping-by-sequencing of highly heterozygous autotetraploid potato. *PLoS One* 8:e62355. 10.1371/journal.pone.0062355 23667470 PMC3648547

[B47] Ukoskit K. Thompson P. G. (1997). Autopolyploidy versus allo-polyploidy and low-density randomly amplified polymorphic DNA linkage maps of sweet potato. *J. Am. Soc. Hortic. Sci.* 122 822–828. 10.21273/JASHS.122.6.822

[B48] Ukoskit K. Thompson P. G. Watson C. E. Lawrence G. W. (1997). Identifying a randomly amplified polymorphic DNA (RAPD) marker linked to a gene for root-knot nematode resistance in sweet potato. *J. Am. Soc. Hortic. Sci.* 122 818–821. 10.21273/JASHS.122.6.818

[B49] Wu S. Lau K. H. Cao Q. Hamilton J. P. Sun H. Zhou C. (2018). Genome sequences of two diploid wild relatives of cultivated sweetpotato reveal targets for genetic improvement. *Nat. Commun.* 9:4580. 10.1038/s41467-018-06983-8 30389915 PMC6214957

[B50] Yamamoto E. Shirasawa K. Kimura T. Monden Y. Tanaka M. Isobe S. (2020). Genetic mapping in autohexaploid sweet potato with low-coverage NGS-based genotyping Data. *G3* 10 2661–2670. 10.1534/g3.120.401433 32482727 PMC7407471

[B51] Yoshida T. (1965). On the geographical distribution of soil nematodes in Chiba Prefecture. *Boll. Chiba Agric. Exp. Stn.* 6 69–78.

[B52] Zhao N. Yu X. Jie Q. Li H. Li H. Hu J. (2013). A genetic linkage map based on AFLP and SSR markers and mapping of QTL for dry-matter content in sweetpotato. *Mol. Breed.* 32 807–820. 10.1007/s11032-013-9908-y

