# Supplementary material for: Mapping of Nematode Resistance in Hexaploid Sweetpotato Using a Next-Generation Sequencing-Based Association Study
Source: Front Plant Sci. 2022 Mar 18;13:858747. doi: 10.3389/fpls.2022.858747 (PMC8972059; doi:10.3389/fpls.2022.858747)
Supplement: Supplementary file 1 [file Data_Sheet_1.DOCX]

Supplementary Material


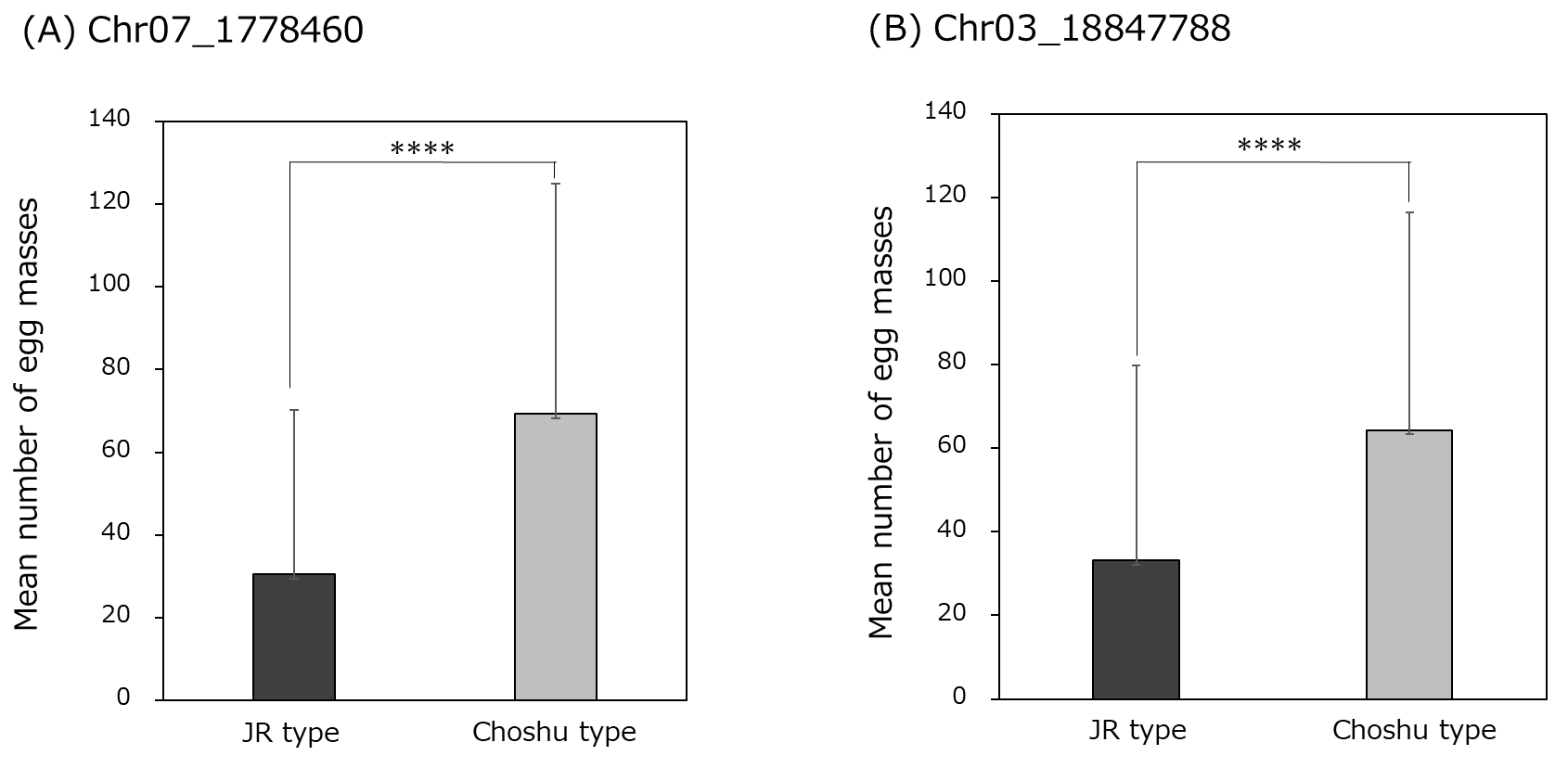


Supplementary Figure 1. The mean number of egg masses on F1 lines grouped according to the genotype of the identified SNPs on Chr03 and Chr07. (A) Chr07_1778460 SNP. (B) Chr03_18847788 SNP.


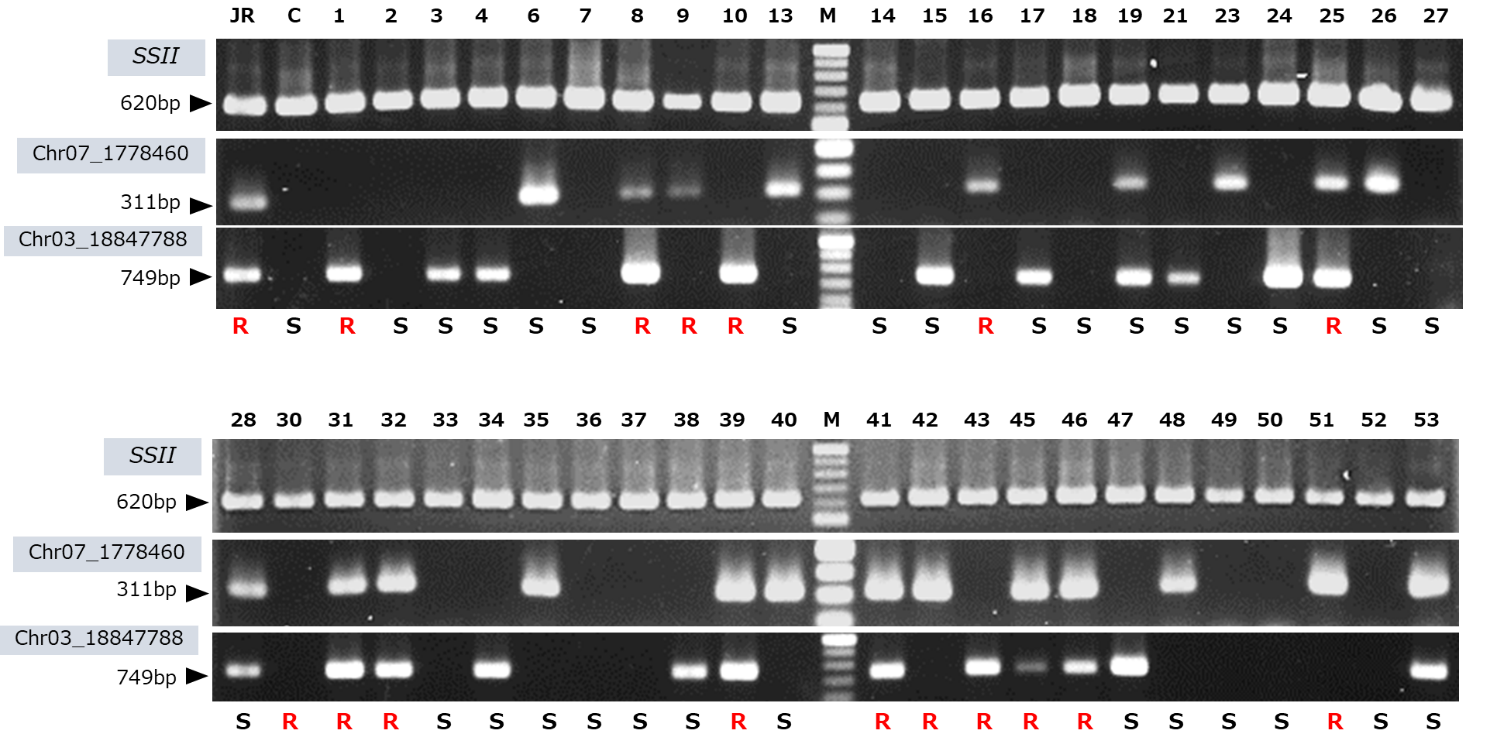


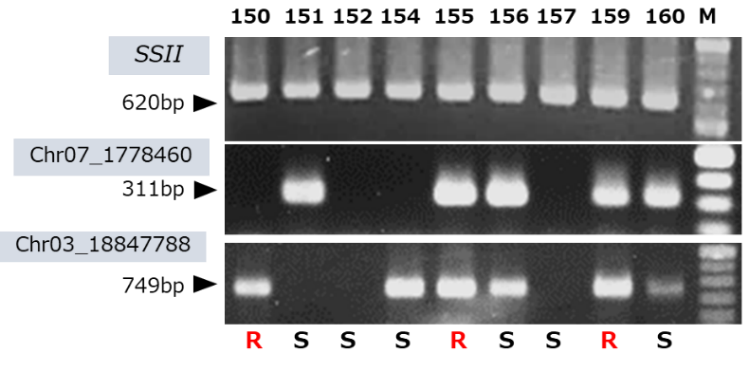

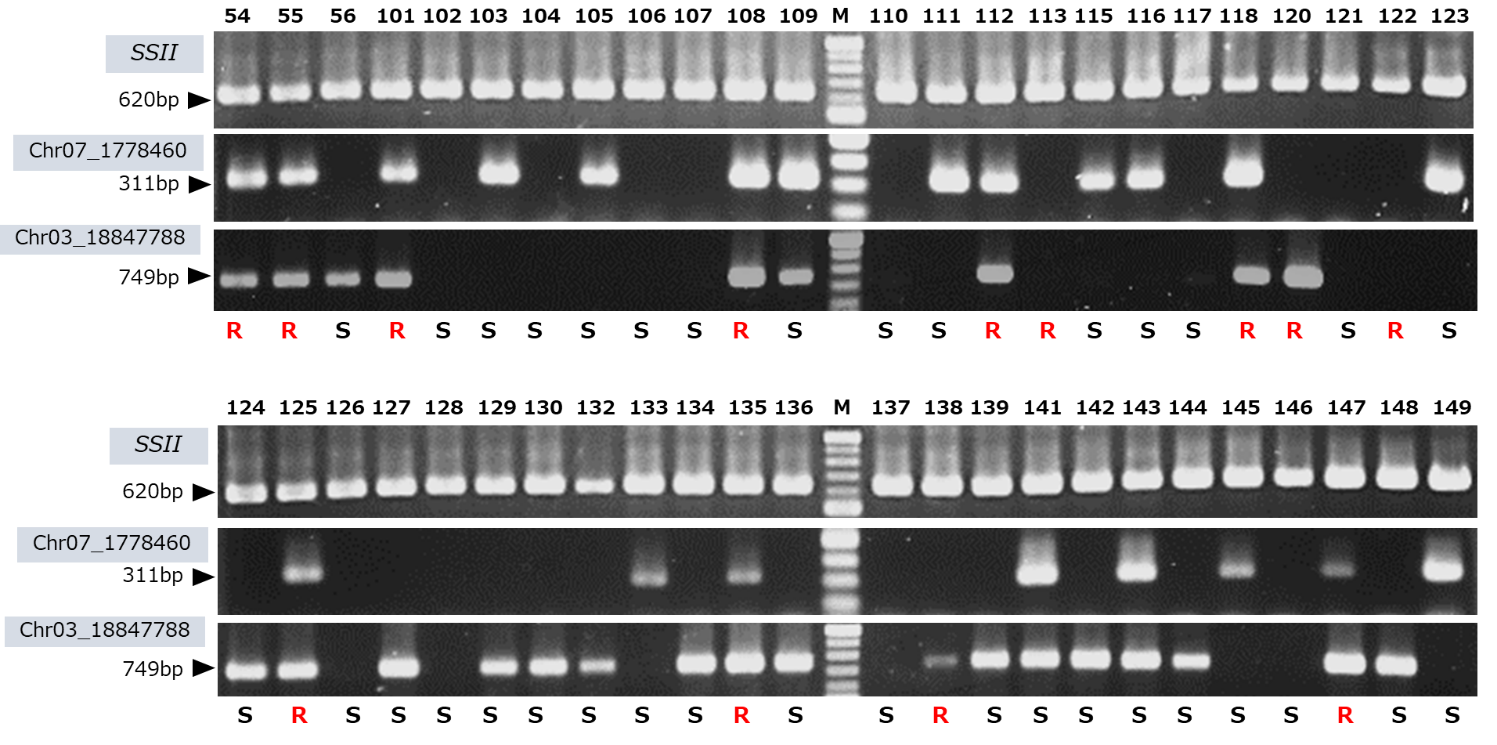


Supplementary Figure 2. Genotyping results using DNA markers derived from the Chr07_1778460 and Chr03_18847788 SNPs of the parental cultivars and 103 F_1_ lines. *SSII*, positive control; JR: J-Red, C: Choshu, 2-160: F1 lines, and M: 100 bp DNA ladder. The evaluation of resistance to the SP2 race is shown on the bottom of each lane. R and S indicate resistance and susceptibility, respectively.


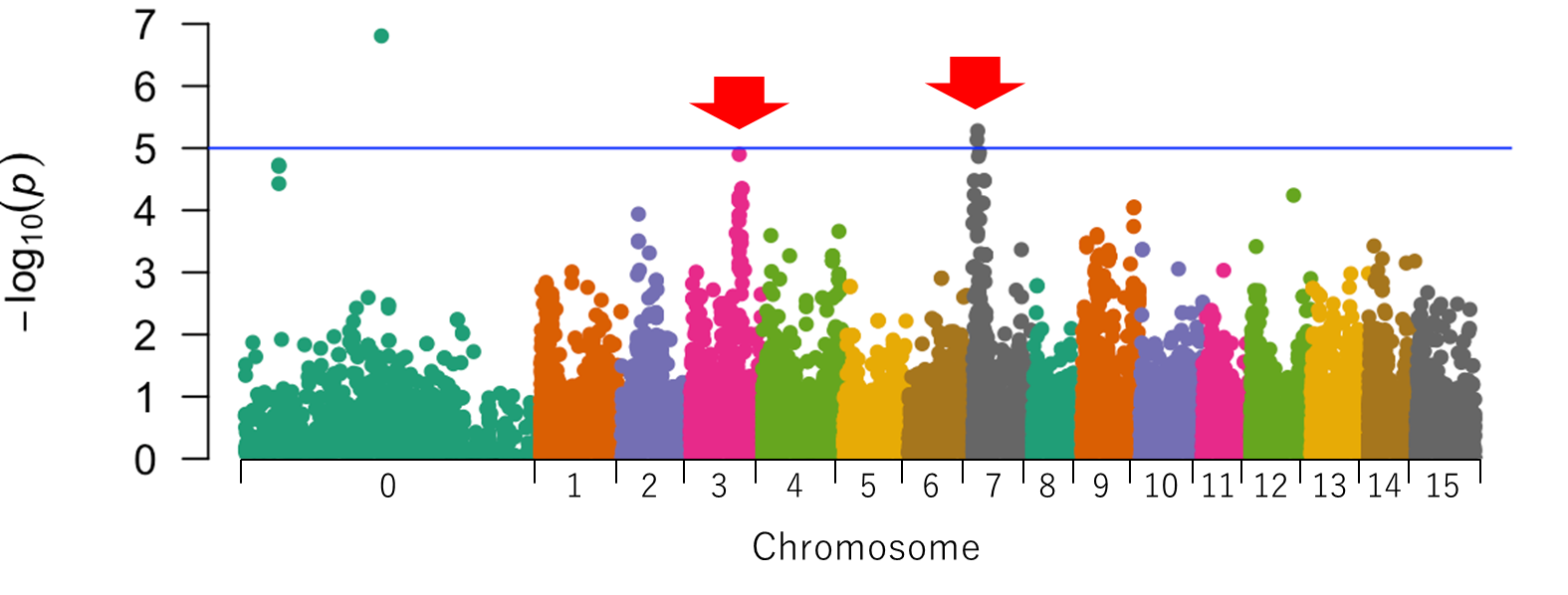
(A)

(B)


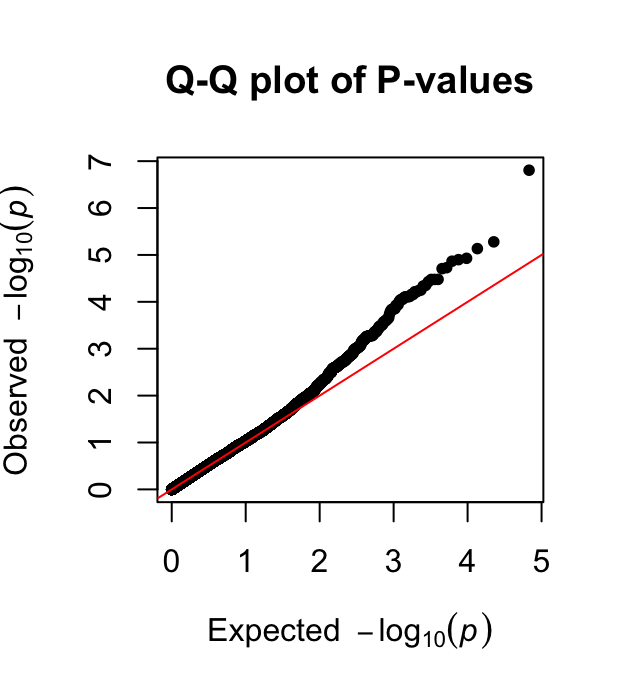


Supplementary Figure 3. Manhattan and Q-Q plots created using only simplex markers. (A) Manhattan plot. X-axis: the chromosomal number; Y-axis: -log_10_ (*P*). The blue line represents the significance threshold –log_10_(1 × 10^-5^) for ‘suggestive’ associations (Turner, 2018). (B) Q-Q plot.
